# No association between rheumatoid arthritis and cognitive impairment in a cross-sectional national sample of older U.S. adults

**DOI:** 10.1186/s41927-021-00198-z

**Published:** 2021-08-18

**Authors:** Michael J. Booth, Mary R. Janevic, Lindsay C. Kobayashi, Daniel J. Clauw, John D. Piette

**Affiliations:** 1 Department of Health Behavior and Health Education, School of Public Health, University of Michigan, 1415 Washington Heights, Ann Arbor, MI 48130 USA; 2 Department of Epidemiology, School of Public Health, University of Michigan, Ann Arbor, MI USA; 3 Department of Anesthesiology, Rheumatology, Psychiatry, School of Medicine, University of Michigan, Ann Arbor, MI USA; 4 Department of Veterans Affairs Center for Clinical Management Research, Ann Arbor, MI USA

**Keywords:** Epidemiology, Rheumatoid arthritis, Cognitive impairment, National health survey

## Abstract

**Background:**

Studies suggest an increased prevalence of cognitive impairment (CI) among people with rheumatoid arthritis (RA). However, most prior studies have used convenience samples which are subject to selection biases or have failed to adjust for key confounding variables. We thus examined the association between CI and RA in a large national probability sample of older US adults.

**Methods:**

Data were from interviews with 4462 participants in the 2016 wave of the nationally representative U.S. Health and Retirement Study with linked Medicare claims. RA diagnoses were identified via a minimum of two ICD-9CM or ICD-10 codes in Medicare billing records during the prior 2 years. The Langa-Weir Classification was used to classify cognitive status as normal, cognitively impaired non-dementia (CIND), or dementia based on a brief neuropsychological battery for self-respondents and informant reports for proxy respondents. We compared the odds of CI between older adults with and without RA using logistic regression, adjusted for age, education, gender, and race.

**Results:**

Medicare records identified a 3.36% prevalence of RA (150/4462). While age, gender, education, and race independently predicted CI status, controlling for these covariates we found no difference in CI prevalence according to RA status (prevalent CI in 36.7% of older adults with RA vs. 34.0% without RA; adjusted OR = 1.08, 95% CI 0.74–1.59, *p* = .69).

**Conclusion:**

There was no association between RA and CI in this national sample of older U.S. adults.

**Supplementary Information:**

The online version contains supplementary material available at 10.1186/s41927-021-00198-z.

## Background

A 2018 systematic review of the risk of cognitive impairment (CI) among people with rheumatoid arthritis (RA) concluded that individuals with RA had significantly greater risk of CI [1]. Effect size analyses suggested that those with RA had significant underperformance in verbal function, memory, and attention [1]. Among the three studies that reported prevalence statistics, the proportion of people with CI in RA ranged from 0 to 31% [2–4]. In contrast, one study reported the prevalence of CI in healthy controls at 7.5% [1, 3]. However, whether these associations extend to the general population of older U.S. adults is unknown.

Researchers have explored several possible reasons why CI may be more prevalent among people with RA. Both depression and chronic pain are common in RA [5, 6], and both conditions are associated with decreased cognitive performance [7–9]. Also, research suggests that some RA treatments may increase the risk of dementia [10]; however, several studies indicate both conventional Disease-Modifying Anti-Rheumatic Drugs (cDMARDs) and anti-TNF biologic DMARDS (bDMARDS) have either no effect or are protective against CI or dementia [4, 11–14]. RA’s peripheral inflammatory processes may also impair cognitive performance [15–18]. Genetic risk factors potentially shared between both RA and dementia have also been hypothesized to contribute to the association, although studies that examined differences between RA and non-RA samples in the frequency of the APOE-e4 allele (a significant genetic risk factor of Alzheimer’s disease) did not find a correlation [18–21].

Not all studies have reported an association between RA and CI. Five population-based studies (not included in the review) using large samples from either registries or insurance databases found negative or null associations between RA and dementia or Alzheimer’s disease (AD) [11, 12, 22–24]. These studies used clinical diagnostic codes to identify RA and CI, and as a consequence, may have misclassified RA, missed subclinical cases of CI, or misclassified dementia/AD [25]. In contrast, studies included in the review with positive findings often used clinic-based convenience samples with systematic assessments of RA and CI and volunteer healthy controls or age-base population norms as a comparator. However, convenience sampling methods may over-represent the prevalence of CI in the RA group and underrepresent CI in the control groups simply due to selection biases. Research examining Mild Cognitive Impairment (MCI) showed that participants recruited from clinics perform more poorly than groups identified via population-based sampling methods [26]. Further, volunteers recruited to “normal” or healthy control groups are likely to have higher cognitive functioning than the general population [26].

The systematic review [1] highlights an additional limitation, specifically that the summed effect size estimates of the association between RA and CI were not adjusted for demographic, clinical, and psychological characteristics of the participants due to differences in measurement or lack of inclusion of these variables in individual studies [1]. Across studies in the review, RA groups tended to be older than controls, which could overestimate the effect of RA on CI [1].

Moreover, the summed effect sizes and many individual studies did not control for education, gender, or race [1]. According to cognitive reserve theory, education is essential for understanding cognitive impairment risk in later life, as more highly educated individuals may maintain cognitive function for longer than those with less education, despite accumulating brain pathology during aging [27]. Research also suggests that lower educational attainment is a risk factor for RA [28]. Therefore, controlling for the confounding effect of education is necessary to understand the RA-CI association.

Another limitation cited in the review is that study samples were predominately female [1]. Though a higher proportion of women than men in RA samples is expected, given that the disease is three times more frequent in women than men [29], in 10 out of the 15 studies included in the review, the samples were between 88 and 100% female. Epidemiological studies show that women have a higher risk, prevalence, rate of decline, and severity of Alzheimer’s disease (AD) [30]. As a consequence, gender may be a confounding variable in the RA-CI relationship.

Research also shows that cognitive impairment risk differs by race, at least in the United States, which was not accounted for in the individual studies nor the summed effect sizes of the review [1, 31]. For example, in non-carriers of the APOE-e4 allele, blacks/African Americans had 2.3 times the risk of AD than whites [31]. Research also shows that blacks/African Americans are less likely to received DMARDs for RA care than whites [32, 33], which could be an additional confounder.

Due to the literature’s lack of clarity, the association between RA and CI requires further epidemiological research. We conducted the current study to provide more definitive information about the potentially increased CI prevalence among people with RA. We used a large sample of older adults with and without an RA diagnosis and cognitive status measured using a validated assessment to determine: (1) whether people with RA had a higher odds of CI than the general population; and (2) whether any differences in CI odds between people with and without RA could be explained by confounding effects of age, gender, educational attainment, or race/ethnicity.

## Methods

### Data sources

The HRS is a nationally-representative longitudinal panel study of US residents 50 years of age and older [34, 35]. Approximately 20,000 participants are surveyed every 2 years. New cohorts are added to the study every 6 years, and participants are followed from entry until voluntary withdrawal or death [34]. The present study sample included respondents surveyed in the 2016 wave of the HRS, the most recent survey year that provides linkable Medicare data. The HRS and the current study including access to sensitive Medicare files was approved by the University of Michigan Health Sciences/Behavioral Sciences Institutional Review Board (HUM00061128, HUM00152177). Informed consent was obtained from all study subjects. No study subjects were under the age of 18. All methods were performed within the relevant confidentiality guidelines and regulations of the Institutional Review Board and the Health and Retirement Study.

The HRS includes information from Medicare-covered health services events for the 78–84% of respondents who authorize linkage across survey years [34]. Medicare billing claims record the reason for a healthcare provider visit listed as International Classification of Diseases, 9th edition, Clinical Modification or 10th edition (ICD-9-CM & ICD-10) codes, Health Care Common Procedure Coding System (HCPCS), and Current Procedural Terminology (CPT-4) codes. To identify HRS respondents with RA, we linked fee-for-service (FFS) Medicare Part A inpatient, outpatient, skilled nursing facility, home health files, and Part B carrier files in the 2 years (2014–2015) preceding the 2016 survey wave. Part C claims, also called Medicare Advantage or Medicare + Choice, are not available for HRS linkage. We addressed differences in respondents’ Medicare enrollment through the exclusionary criteria discussed below.

### Sample eligibility

From the initial 20,890 HRS respondents in 2016, we excluded those who were Medicare-ineligible or who did not consent to Medicare linkage (*n* = 12,046 excluded). To avoid missing data biases affecting the availability of RA diagnoses, we further excluded those with Medicare linkage who did not have full FFS parts A & B coverage from 2014 to 2015, defined as 11 months or more per year (*n* = 4382 excluded). This last step excluded anyone with Part C claims greater than 1 month per year, leaving a total of 4462 respondents in our final sample. We did not exclude respondents between the ages of 50–64 who had Medicare benefits due to disability, end-stage-renal disease (ESRD), or amyotrophic lateral sclerosis (ALS).

### Identifying RA

The validity of identifying RA via ICD code-based algorithms varies by the population under consideration and the methods used. A systematic review of ICD-9 code-based algorithms for the detection of RA in administrative databases found that the highest positive predictive values (PPV; the proportion of true positives out of all algorithm-identified positives) come from algorithms that include a minimum of two RA diagnostic codes and additional information related to whether a rheumatologist made the claim or if the RA patient received DMARDs, the most common class of medications for people with RA [36]. However, additional requirements for RA classification come with generalization limitations and tradeoffs. For instance, over the course of 2 years, approximately 34% of people with RA see a rheumatologist at least once [37]. Further, estimates from two population based studies showed that less than half of people with RA had associated DMARDs prescriptions [32, 37]. Though additional requirements of having a rheumatologist make the claim, or including DMARD prescriptions increases the PPV of an algorithm, these requirements also identify specific and narrow RA populations that are unlikely to represent all adults with RA. Therefore, we conducted our analysis using an algorithm that is likely to include the most people with RA, and then conducted two sensitivity analyses with increasing PPV’s but higher restrictions, discussed in more detail below.

We identified cases of RA via participants’ Medicare claims by requiring a minimum of two billing diagnoses of ICD-9CM codes 714* or ICD-10 codes M05*or M06*, between study years 2014–2015. We included any code listed either as the principal diagnosis or in one of the 25 primary/secondary diagnostic fields from Medicare Part A files or the 12 fields from routine clinical visits in Part B carrier files. Claims had to be more than 1 day apart. We excluded claims from non-licensed health care providers, such as durable medical equipment providers and ambulance services.

For the sensitivity analyses, we applied identical methods described above using 1). an algorithm requiring, in addition to two RA claims, a minimum of one from a rheumatologist, and 2) a different algorithm requiring two RA codes from any provider, and one DMARD prescription. We identified rheumatology clinic-based claims using CMS provider specialty code “66” listed in at least one of the 13 specialty billing fields in the Part B carrier files. We identified DMARDs using generic names (see appendix) from Medicare part D summary files in 2014–2015.

### Measurement of cognitive impairment

HRS respondents’ cognitive status was measured with the Langa-Weir Classification [38]. The Langa-Weir measure provides a 27-point scale of cognition for self-respondents (the modified Telephone Interview for Cognitive Status; or TICS-m) and an 11 point scale for proxy respondents, representing cognition at the time of the 2016 HRS interview [38, 39]. The use of proxy respondents in the HRS allows people who are either physically or cognitively incapable of completing the survey to participate, which ensures adequate representation of the older adult population and reduces bias related to study attrition from low levels of cognitive ability [39, 40]. In 2016, proxies represented 4.5% of all HRS respondents and 3% of those with CIND or dementia.

The Langa-Weir classification assesses cognitive function for self-respondents in the HRS using an adapted version of the Telephone Interview for Cognitive Status (TICS). The adapted TICS consists of immediate and delayed 10-noun free recall (respondents immediately recall a list of 10 words, then remember the list after a delay) and serial 7’s subtraction tests (respondents subtract seven from 100, then continue to remove 7 five more times) to assess memory, and backward count to evaluate attention and processing speed [38, 39, 41]. Proxy measures of CI include caregivers’ assessment of the person’s cognition in the areas of memory (excellent, very good, good, fair, or poor) and instrumental activities of daily living limitations (IADL, scored 0–5). The proxy measure also includes the trained interviewer’s overall estimation of risk for CI (No CI, may have CI, has CI) [38].

The Langa Weir classification yields three groups: normal cognition, “cognitive impairment non-dementia” (CIND), or dementia [42]. Cut points in the Langa-Weir classification produced the same population distribution of cognitive ability as estimated in the Aging, Demographics, and Memory Study (ADAMS), a subsample of HRS respondents who underwent extensive neuropsychological testing and clinical assessment [38, 43]. Using the 27-point self-report scale, cut points for the three categories are normal cognition (12–27), CIND (7–11), and dementia (0–6) [38]. For the 11-point proxy scale, cut points are normal (0–2), CIND (3–5), and dementia (6–11). Both the self-report and proxy scores use imputed values for missing data [38]. Documentation of imputation methods for all cognitive measures is available at the HRS website [44]. Due to low counts of dementia in the RA group, we collapsed the Langa-Weir classification into a binary yes-or-no variable with normal cognition versus CIND or dementia.

### Covariates

Additional variables include respondent age at the time of the survey, educational attainment (less than high school, high school graduate/GED, any college or more), gender (male/female), and race (White/Non-White).

### Statistical methods

We calculated differences in the sociodemographic characteristics of subgroups with and without RA and the proportion of respondents in each group with CI, defined as CIND or dementia per the Langa-Weir classification. We then examined the relative odds of cognitive impairment among people with RA versus no RA using unadjusted and adjusted logistic regression using RA as the predictor of interest and CI as the outcome. The adjusted model included age (centered at its mean), educational attainment, gender, and race as controls for confounding.

The HRS uses a national probability sample and provides the appropriate weights for complex survey design analysis and national estimates. Our criteria requiring 2 years of complete FSS linked Medicare parts A & B claims reduced the original HRS sample by 79%. This reduction in sample size resulted in having no population members from 24 out of the 80 strata from which HRS samples are drawn (strata in the HRS are non-overlapping Metropolitan Statistical areas, single counties, or groups of small counties used to stratify the population). Because our reduced sample was not nationally representative, and we could not determine if our sample’s weighting reflected the original study’s probability distribution, we did not employ survey design weighting in our analysis. We performed all analyses using STATA 16.1 MP (College Station, TX).

## Results

### Population characteristics (Table 1)

One hundred and fifty out of 4462 eligible HRS respondents were classified as having RA (3.36%), slightly above the population prevalence of RA in this age group reported elsewhere [29]. Those with RA were, on average, 1.7 years younger (75.8 years of age, SD 7.9 vs. 77.5 years, SD 8.2, *p* = .008) than those without RA, and more often female (76.0% female vs. 24.0% male, p = <.001). The RA group had a higher proportion of non-Whites than Whites (27.3% for RA vs. 16.9% for non-RA, *p* < .001), and lower educational attainment (24% less than high school for RA vs. 17.0% for non-RA, *p* = .015).

**Table 1:** Population Characteristics of RA and Non-RA Respondents, the Health and Retirement Study 2016 Wave

Population characteristic	RA positive (*n* = 150)	RA negative (*n* = 4312)	*p*
Age 2016 (mean, SD), years	75.8 (SD 7.9)	77.5 (SD 8.2)	*.008
Sex (n, %)			* < .001
Male	36 (24.0%)	1732 (40.2%	
Female	114 (76.0%)	2580 (59.8%)	
Race			* < .001
White/Caucasian	109 (72.7%)	3582 (83.1%)	
Non-White	41 (27.3%)	730 (16.9%)	
Education (n, %)			*0.015
Less than High School	36 (24.0%)	734 (17.0%)	
High School or Equivalent	86 (57.3%)	2392 (55.5%)	
Any College or More	28 (18.7%)	1186 (27.5%)	

Continuous measures tested with t-test of equal variance

Categorical measures tested using Pearson’s Chi Square

*RA* Rheumatoid Arthritis

*Denotes statistically significant *p*-value at .05 alpha

### Principal analysis (Table 2)

Of the 150 respondents with RA, 95 (63.3%) had normal cognition at the time of their survey, 37 were classified as CIND (24.7%), and 18 as dementia (12.0%). Of the 4312 respondents without RA, 2848 (66.1%) had normal cognition, 961 were classified as CIND (22.3%) and 503 as dementia (11.7%). In unadjusted analyses, people with RA were no more likely to have CI than the general population (OR = 1.12, 95% CI 0.80–1.58, *p* = 0.49). Adjusting for sociodemographic covariates affected the association (adjusted OR [AOR] = 1.08, 95% CI 0.74–1.59, *p* = 0.69), age (reference mean age 77.5 years, AOR = 1.10, 95% CI 1.09–1.11, *p* < 0.001), education (reference any college: high school AOR = 2.63, 95% CI 2.17–3.16) *p* < .0001; less than high school OR = 8.81, 95% CI 7.03–11.06, p < 0.001), gender (female AOR = 0.84, 95% CI 0.73–0.96, *p* = 0.014), and race (non-White AOR = 2.62, 95% CI 2.18–3.17, *p* < .001).

**Table 2:** Odds ratios and 95% confidence intervals of cognitive impairment in rheumatoid arthritis

Predictors	Unadjusted model	*p*	Adjusted model	*p*
Rheumatoid Arthritis	1.12 (.80-1.58)	0.49	1.08 (.74-1.59)	0.69
Age (years, centered)			1.10 (1.09-1.11)	*<.001
Gender (reference male)				
Female			.84 (.72-.96)	*.014
Race (reference White)				
Non-White			2.62 (2.18-3.17)	*<.001
Education (reference any college)				
High School or Equivalent			2.63 (2.17-3.16)	*<.001
Less than High School			8.81 (7.03-11.06)	*<.001

*Denotes statistically significant *p*-value at .05 alpha

### Sensitivity analyses

We performed the sensitivity analysis using two alternative definitions of RA as described previously. The algorithm for a minimum of two diagnoses, a minimum of one of which was from a rheumatologist, classified 77 of the 4462 respondents with RA (1.72%). The unadjusted OR for CI was .77 (95% CI .47–1.27, *p* = .308), and the adjusted OR was .87 (.50–1.52, *p* = .63). The second sensitivity analysis requiring two RA codes from any provider, and at least one DMARD prescription reduced the study population to respondents who also had Part D coverage (*n* = 2846), which reduced the number of people with RA (*n* = 48, 1.69%). The unadjusted OR was 1.30 (.73–2.31, *p* = .38) and adjusted OR 1.27 (.65–2.47, *p* = .48).

To verify that our primary results were not due to the thresholds for defining CI in the Langa-Weir classification, we ran adjusted and unadjusted ordinary least squares regression models with the 27-point cognitive impairment scale as a continuous outcome and RA as the predictor (the 27 point-scale does not include proxy respondents). In the continuous measure, we found no difference in the effect of RA on cognitive performance compared to those without RA (results available in supplementary materials).

## Discussion

Using a large sample of older US adults, an algorithm likely to capture the full range of people with RA, and a validated measure of cognitive impairment, we found no increased CI risk among people with RA relative to comparable subjects without the disease. In our study, the prevalence of CI among people with RA was approximately 36.7% (combining CIND and dementia), which is within the range of previously cited studies. However, using a large population-based control group of people without RA, we found no association either in the unadjusted odds of CI, or the odds when adjusting for age, education, gender, and race. These findings are in alignment with other population-based studies that found no difference in CI by RA status but contrast with the many clinic-based samples that found a positive association. Our research addresses limitations in previous studies related to convenience sampling and controlling for the confounding effects of age, education, gender, and race. However, our study has several limitations of its own, as discussed below.

There are inherent weaknesses in the use of ICD diagnostic codes to detect RA. Coding errors may result from poor patient-physician communication, differences in coder expertise or a clinician’s knowledge of the illness, intentional and unintentional recording errors, or discrepancies between electronic and written records [45]. Further, because we do not have a validated gold standard diagnosis of RA to detect cases, our exposure group may contain people without RA and control group people with RA, which would bias our results towards the null. Such limitations arise from the unavoidable trade-offs of using national survey data rather than clinic-based samples. Though the HRS includes self-reported RA, our previous research showed a very low PPV of self-reported RA when compared against Medicare records (PPV = .05–.16 across three algorithms) [46]. Therefore, the use of Medicare based algorithms to detect RA in the HRS in the most valid method available. Looking at our RA group’s characteristics, we found that participants classified as having RA had, on average, 11.3 billing claims for RA-related care in the 2 years before their survey. The gender ratio was 3 to 1 female to males, which also follows the disease’s typical distribution [29]. Our sensitivity analyses using two different detection algorithms likewise did not find an increased odds of CI in RA.

Our findings are limited by the cross-sectional design. The reduced sample and inability to use sample weights also limits our ability to generalize to the entire U.S. population. Nonetheless, the HRS sample is selected to be nationally representative and avoids the selection bias inherent in clinic-based or smaller surveys.

Our sensitivity analyses results, though not statistically significant, show that RA classification may affect the associations between RA and CI observed in population-based studies. Using only two RA diagnoses resulted in an adjusted OR very close to 1.0. This may mean that the algorithm does not adequately distinguish between people with and without RA, since research suggests the PPV of 2 RA codes is low [36]; however, the algorithm also captures a broader range of people with RA compared to stricter methods. Requiring a rheumatologist’s diagnosis resulted in an AOR below 1.0 and requiring DMARDs above 1.0. Though some variability is expected, these changes around the null hypothesis indicate that the RA-CI association may be sensitive to who is classified as RA in administrative data, which may influence results in larger samples with more statistical power to detect small differences.

Our study suggests that caution is needed in interpreting prior research that suggests an increased prevalence of CI in RA. The results of our study indicate that there is no significant difference in the risk of CI between people with RA and the general population, and confirms that age, gender, education, and race are confounding variables in the RA-CI relationship. We recommend that future studies reduce potential bias created from convenience sampling and use longitudinal rather than cross-sectional study designs to better understand the link between RA and CI. We also recommend that future studies using matched case-control designs match on age, education, gender, and race (to control for confounding), or when comparing RA to the general population control at minimum for age, education, gender, and race. Future population-based research on the RA-CI association should also consider a range or possible RA detection methods, and the tradeoffs resulting from each.

## Supplementary Information

**Additional file 1.**

**Additional file 2.**

## Data Availability

The data that support the findings of this study are available from the Health and Retirement Study, but restrictions apply to the availability of these data, which were used in a protected virtual desktop infrastructure maintained by the University of Michigan Institute for Social Research. The Stata .do files used to manage and analyze these data are available from the corresponding author upon reasonable request and will recreate this study within the restricted Health and Retirement Study computing environment. No sharing of the data is permitted. Reporting of the data in this study was subject to review and disclosure limitations by the Health and Retirement Study.

## Abbreviations

AD: Alzheimer’s disease
ADAMS: Aging demographics and memory study
AOR: Adjusted odds ratio
bDMARDs: Biologic disease-modifying anti-rheumatic drugs
CI: Cognitive impairment
cDMARDs: Conventional disease-modifying anti-rheumatic drugs
CIND: Cognitive impairment, non-dementia
CMS: Center for Medicare & Medicaid services
CPT-4: Current procedural terminology codes
DMARD: Disease-modifying anti-rheumatic drug
FFS: Fee for service
HCPCS: Health care common procedure coding system
HRS: Health and retirement study
ICD-9-CM: International classification of diseases, clinical modification, 9th edition
ICD-10: International classification of diseases, 10th edition
MCI: Mild cognitive impairment
TICS: Telephone interview for cognitive status
tsDMARD: Targeted synthetic disease-modifying anti-rheumatic drug
OR: Odds ratio
PPV: Positive predictive value
RA: Rheumatoid arthritis
