# Supplementary material for: No association between rheumatoid arthritis and cognitive impairment in a cross-sectional national sample of older U.S. adults
Source: BMC Rheumatol. 2021 Aug 18;5:24. doi: 10.1186/s41927-021-00198-z (PMC8371766; doi:10.1186/s41927-021-00198-z)
Supplement: Supplementary file 1 — Additional file 1. [file 41927_2021_198_MOESM1_ESM.docx]

**No Association Between Rheumatoid Arthritis and Cognitive Impairment in a Cross-Sectional National Sample of Older U.S. Adults**

Michael J. Booth, MS^1^; Mary R. Janevic, PhD^1^; Lindsay C. Kobayashi, PhD^2^; Daniel J. Clauw, MD^3^; John D. Piette, PhD^1,4^

^1^ Department of Health Behavior and Health Education, School of Public Health, University of Michigan, Ann Arbor, Michigan, USA

^2^ Department of Epidemiology, School of Public Health, University of Michigan, Ann Arbor, Michigan, USA

^3^ Department of Anesthesiology, Rheumatology, Psychiatry, School of Medicine, University of Michigan, Ann Arbor, Michigan, USA

^4^ Department of Veterans Affairs Center for Clinical Management Research, Ann Arbor, Michigan, USA
