# Supplementary material for: No association between rheumatoid arthritis and cognitive impairment in a cross-sectional national sample of older U.S. adults
Source: BMC Rheumatol. 2021 Aug 18;5:24. doi: 10.1186/s41927-021-00198-z (PMC8371766; doi:10.1186/s41927-021-00198-z)
Supplement: Supplementary file 2 — Additional file 2. [file 41927_2021_198_MOESM2_ESM.docx]

There were 4,155 total HRS self-respondents (non-proxy), of which 142 were classified with RA. On the 27-point scale of cognition for self-respondents, those with RA performed no differently than those without RA (unadjusted coefficient -0.42, 95% CI = -1.2 - 0.36, p=0.29, adjusted coefficient -0.10, CI = 0.78 - .57, p=0.77). In the adjusted model, age (-0.17, CI = -0.19 - -0.16, p<.001), female gender (0.64, CI = 0.39 - 0.8, p*<.001), non-white race (-2.26, CI = -2.60 - -1.92, p<.001), high school or equivalent (-1.95, CI = -2.23 - -1.66, p<.001), and less than high school education (-5.21, CI = -5.61 - -4.82, p<.001) independently predicted cognitive performance.

Rheumatoid Arthritis and a 27-Point Scale of Cognition, the Health and Retirement Study, 2016

Independent Variables 27-Point Cognition Scale Coefficient and 95% CI

| n=4,155 | Unadjusted | *P* | Adjusted | *P* |
| --- | --- | --- | --- | --- |
| Rheumatoid Arthritis | -0.42 (-1.2 - 0.36) | 0.29 | -0.10 (0.78 - 0.57) | 0.77 |
| Age (years, centered) |  |  | -0.17 (-0.19 - -0.16) | *<.001 |
| Gender (reference male) Female |  |  | 0.64 (0.39 - 0.89) | *<.001 |

Race (reference White)

Non-White -2.26 (-2.60 - -1.92) *<.001

Education (reference any college)

| High School or Equivalent | -1.95 (-2.23 - -1.66) | *<.001 |
| --- | --- | --- |
| Less than High School | -5.21 (-5.61 - -4.82) | *<.001 |

* Denotes statistically significant p-value at .05 alpha CI = Confidence Interval
